# Disrupted food systems in the WHO European region – a threat or opportunity for healthy and sustainable food and nutrition?

**DOI:** 10.1007/s12571-020-01079-y

**Published:** 2020-07-23

**Authors:** Holly L. Rippin, Kremlin Wickramasinghe, Afton Halloran, Stephen Whiting, Julianne Williams, Kathrin Hetz, Adriana Pinedo, João J. Breda

**Affiliations:** 1 World Health Organization European Office for the Prevention and Control of Noncommunicable Diseases, Moscow, Russian Federation; 2 Department of Nutrition, Exercise and Sports, University of Copenhagen, Frederiksberg, Denmark

**Keywords:** Healthy diets, Sustainable diets, Noncommunicable diseases, Food systems, COVID-19, WHO European region

## Abstract

Dietary health and sustainability are inextricably linked. Food systems that are not sustainable often fail to provide the amount or types of food needed to ensure population health. The ongoing pandemic threatens to exacerbate malnutrition, and noncommunicable diseases (NCDs). This paper discusses threats and opportunities for food environments and health status across the WHO European Region in the current context . These opportunities and threats are focused around four key areas: NCDs and health systems; dietary behaviour; food insecurity and vulnerable groups; and food supply mechanisms. Food systems were already under great stress. Now with the pandemic, the challenges to food systems in the WHO European Region have been exacerbated, demanding from all levels of government swift adaptations to manage healthiness, availability, accessibility and affordability of food. Cities and governments in the Region should capitalize on this unique opportunity to ‘build back better’ and make bold and lasting changes to the food system and consequently to the health and wellbeing of people and sustainability of the planet.

## Introduction

Dietary health and sustainability are inextricably linked. Food systems that are not sustainable fail to provide the amount or types of food needed to ensure population health. The current global situation is significantly challenging food systems at supranational, national and local levels. It threatens to exacerbate the current health crisis by increasing the problem of malnutrition, particularly in the form of noncommunicable diseases (NCDs). Within the WHO European Region NCDs (cardiovascular diseases, cancers, chronic respiratory diseases and diabetes) are the biggest causes of morbidity and mortality. Overweight and obesity – a major NCD risk factor – affected 59% of adults in the WHO European Region in 2016 (WHO 2020).

How are food environments and health status across the WHO European Region transforming during the pandemic, and how will they continue to change after response measures have been lifted and in the months and years to come? Millions across Europe and the world are experiencing lockdown restrictions of varying severity, drastically changing the way we live, including our dietary and health-related behaviours. The future consequences of these changes are uncertain, but initial evidence shows that these measures contradict established public health advice, particularly regarding eating healthily and sustainably, and being active.

### NCDs and health systems

The impacts of the COVID-19 pandemic, together with the public health outcomes of widespread lockdown measures, create a perfect storm for NCD risk factors including obesity and poor diet, physical inactivity, alcohol consumption and tobacco use. In Italy, 99% of deceased patients with COVID-19 suffered co-morbidities, primarily NCDs, and there is evidence for this elsewhere across Europe and globally (Istituto Superiore di Sanità 2020; Instituto de Salud Carlos III 2020; China CDC Weekly 2020; CDC COVID-19 Response Team 2020). Evidence also suggests that body mass index (BMI) is positively associated with worse outcomes (Peng et al. 2020). COVID-19 can present more severe symptoms in people with obesity-related conditions, due to the increased risk of obesity-related NCDs such as diabetes (Hussain et al. 2020). In many WHO European Region countries, more than 50% of adults are overweight or obese, putting them in a particularly vulnerable position (WHO 2014a).

Further, individuals with obesity experience stigma and may suffer higher rates of mental health issues (Emmer et al. 2020), which may be exacerbated by self-isolation. The link between COVID-19 and NCDs means that despite the apparent immediate severity of this pandemic, its long-term health consequences have the potential to outweigh the short-term impact. Health systems and society therefore need to fight both risk factors for and bias against overweight and obesity in order to improve health outcomes in a sustainable manner. If used correctly, the pandemic could represent an opportunity to shift towards a health system that better fulfills the role of health promoting settings. It could provide the impetus needed to move from a system built around retrospective reaction, to one that is proactive and based on prevention and promotion of wellbeing in a holistic fashion – highly relevant in a Region with high NCD prevalence. In the immediate and short-term, health services should continue to provide essential nutrition services for vulnerable groups including pregnant women, newborns, the elderly and sick children. They should also provide appropriate support for mothers to breastfeed, including those with COVID-19, and communicate accurate information on maternal, infant and young child nutrition, particularly on complementary feeding. A modern and coherent food systems approach must be conducive to food and nutrition-related behaviors that contribute to a more efficient health system by reducing the burden of diet-related diseases.

### Dietary behaviour

Lockdown measures have shifted the dynamics of dietary behaviour. Many national governments in the Region forced the closure of non-essential services for an extended period, including food provision businesses such as restaurants and cafes. This could impact population dietary habits in various ways, both positive and negative, in the immediate and medium to long-term. People may engage in more home gardening and cooking out of necessity and pleasure, potentially leading to better quality diets (Nielsen 2020). This may also have a beneficial impact on mental health. Conversely, lack of routine caused by restricted movement may encourage a ‘holiday eating’ pattern, potentially resulting in over-consumption (Venema 2020). However, whether these trends are playing out at all socioeconomic levels is also unclear. It is also uncertain as to whether these new patterns will be sustained as lockdown measures are relaxed and the out-of-home food sector reopens.

Alternatively, individuals may opt for food delivery rather than venturing outside to purchase food at supermarkets or fresh markets. Even pre-pandemic, increasing numbers of urban-dwellers in the Region were purchasing from online food delivery services due to time scarcity, a major inhibiting factor in the preparation of home-cooked meals. A pre-COVID-19 non-representative survey of 10 European countries found that every fifth meal was consumed outside of the home, of which 80% were from commercial outlets (IRi 2020). Many of these convenience options are high in fat, saturated fat, sugar and salt and are associated with NCD risk. However, lack of supermarket visits may reduce the bulk purchase of such unhealthy, ultra-processed foods and limit exposure to in-store promotions that might otherwise encourage over-consumption. While these adjustments may be temporary, evidence from previous intense social transformations shows that times of crisis can accelerate transformation in the food system, for better or for worse. There exists an opportunity to harness and encourage healthier cooking and eating, both at home and within the out of home sector as this reopens. Communication and multisectoral collaboration will be required to facilitate this across all socioeconomic levels, ensuring that no population group is left behind.

Restricted movement and temporary business closures are affecting normal food-related practices. In a globalized food system, limited access to fresh foods due to panic buying, stockpiling and food shortages may compromise the ability to eat a balanced diet and increase consumption of highly processed foods, often high in fats, sugars and salt (World Economic Forum 2020; World Health Organization 2018). Good nutrition is crucial for health, particularly in times when the immune system might need to fight disease.

### Food insecurity and vulnerable groups

School closures negatively affect the food environment and possibilities for physical activity among children (World Food Programme 2020). Among these effects are greater risk of food insecurity, as well as sedentary behaviours, both of which are linked to childhood overweight and obesity (Rundle et al. 2020). Schools can play a vital part in mitigating the risks of both over and undernutrition, as there is evidence that food insecurity increases during school holidays, particularly for vulnerable groups (Graham et al. 2016). The health and economic implications of this crisis are intertwined. In the wake of the pandemic, many face job insecurity and reduced income, and the economic costs of social distancing tend to hit the poorest, most vulnerable and marginalized members of society. There have been reports of charities tackling such food insecurity, like food banks, experiencing higher demand across the WHO European Region. Over 80% of European food banks have seen increased requests for food aid and emergency food assistance (European Food Banks Federation 2020). Healthy and sustainable food systems must also serve these vulnerable populations. Increased unemployment and financial insecurity may also lead to elevated alcohol and tobacco consumption, worsening NCD risk and outcomes (WHO 2014b; Henkel 2011).

Further, the elderly population and those with compromised immune systems across the WHO European Region have been specifically instructed to strictly self-isolate for an extensive period. There are particularly strong associations of COVID-19 incidence, hospitalization risk and poorer outcomes in the elderly and those with diabetes, obesity and hypertension. Older people may face unique food and nutrition challenges, especially in contexts of isolation and/or poverty (Starr et al. 2015). Elderly care homes are at high risk of residents contracting COVID-19, and there is evidence that deaths in care homes are increasing in some countries at alarming rates (Office for National Statistics 2020). Additionally, older populations are often not adequately considered when food systems and nutrition issues are discussed. This double burden may put this group at particular risk of poorer outcome for COVID-19 and NCDs.

### Food supply mechanisms

The supply side of the food system is also impacted by COVID-19 (Torero 2020). Labour and logistics capacity issues mean that companies are struggling to meet consumer demand for fresh food (CBI Ministry of Foreign Affairs 2020). Restricted movement has prevented migrant seasonal workers from picking fruit and vegetables, resulting in food loss and waste, and potentially food safety risks. No food can be considered healthy if it is unsafe to eat, and it is important that neither health nor environmental sustainability are compromised due to spoiled food or increased food loss and waste.

The COVID-19 crisis may provide an opportunity to reassess our diets and food systems and to explore ways to add diversity. Traditional and regional diets could play a significant role in responding to local and national challenges in ensuring healthy and sustainable diets, by promoting sustainable farming practices and smarter and shorter food supply chains to buffer against the multiple shocks created in times of crisis. A move towards such diets could also provide an opportunity for individuals to reflect and adapt their dietary choices related to food access and consumption. Investment initiatives aimed at maintaining food systems as part of the COVID-19 response and beyond should be an integral part of helping individuals and society recover better, both from a nutritional and an environmental perspective.

## Conclusion

The current pandemic poses significant challenges to food systems in the WHO European Region, demanding from all levels of government, particularly cities, swift adaptations to manage the healthiness, availability, accessibility and affordability of food. There is no ‘business as usual’ – we cannot afford to ignore the issues raised in this think piece. Food security and nutrition are emerging as one of the biggest concerns as a collateral effect of the pandemic. Immediate action is needed to address, monitor and understand this changing situation. Governments in the Region should capitalize on this unique opportunity to ‘build back better’ and make bold and lasting changes to the food system and consequently to people’s health and wellbeing. Work is urgently needed to gain a better understanding of and to help countries navigate this rapidly changing landscape. This includes a need to innovate surveillance systems to track the impact and to anticipate mitigation measures in terms of food systems for populations and at-risk subgroups in urban and rural contexts. We must leverage these opportunities to contribute to a more sustainable environment and to future proof against the next pandemic.

## Data Availability

All data and material are appropriately referenced.
